# Acute Immune Thrombocytopenia Following Influenza Vaccination in a Patient With Untreated Helicobacter pylori Infection

**DOI:** 10.7759/cureus.43946

**Published:** 2023-08-22

**Authors:** Edgar Asiimwe, Kanwarpal S Kahlon

**Affiliations:** 1 Internal Medicine, University of California Los Angeles, Los Angeles, USA; 2 Hematology and Oncology, University of California Los Angeles, Los Angeles, USA

**Keywords:** immune-mediated thrombocytopenia, adverse effects of vaccines, immune thrombocytopenia (itp), influenza vaccine, h.pylori infection

## Abstract

A 70-year-old man with previously normal comprehensive blood counts (CBCs) was referred to our hospital for acute thrombocytopenia. Following a negative workup for secondary causes, we diagnosed immune thrombocytopenia (ITP). Aside from the influenza vaccine administered six days before presentation, there was no discernable precipitant on history. His only risk factor for ITP was untreated *Helicobacter *​​​​​​*pylori *diagnosed over two months prior. With treatment, the patient’s platelets normalized within three days. ITP following influenza vaccination has been documented in the literature and reported to regulatory bodies. Our case indicates that individuals with untreated* H. pylori* infection might be particularly vulnerable to such occurrences.

## Introduction

Immune thrombocytopenia (ITP) is a common cause of isolated, asymptomatic thrombocytopenia [1]. While the pathophysiology of the disease is not entirely understood, the prevailing theory is that of molecular mimicry whereby B-cells, in response to some antigen, produce antibodies with a simultaneous affinity for their host’s platelets [1]. The stimulus for this aberrant immunologic response remains a mystery in the majority of cases (primary ITP); however, in a considerable minority (secondary ITP), a precipitant is identified and can be extrinsic, such as with infection (e.g., HIV, *Helicobacter pylori*, etc.), or drugs (e.g., quinine and certain vaccines), or intrinsic as with systemic autoimmune diseases, for example, systemic lupus erythematous (SLE).

ITP is a diagnosis of exclusion, and other causes of thrombocytopenia must be ruled out in the patient presenting with isolated thrombocytopenia [1]. In investigating new-onset thrombocytopenia, most clinicians follow a framework that can be distilled into three main tasks (once pseudothrombocytopenia has been ruled out): (1) investigation of decreased bone marrow production (assessed by reviewing the comprehensive blood count [CBC] for additional cytopenias); (2) investigation of consumptive processes, such as sepsis, thrombotic microangiopathy (TMA), and diffuse intravascular coagulation (DIC); and (3) investigation of active sequestration (usually splenic as seen in cirrhosis). In addition, in the appropriate clinical context, underlying hematologic malignancy is investigated. When alternative causes for isolated thrombocytopenia have been ruled out, the diagnosis of ITP is established.

We do not fully understand why some patients, especially those without a history of autoimmune disorders, develop ITP. Nonetheless, observational studies have identified some risk factors, such as age and *H. pylori* infection [2-4]. This report describes a case of a 70-year-old male patient with untreated *H. pylori *infection who developed ITP after receiving the influenza vaccine.

## Case presentation

**Figure 1:**
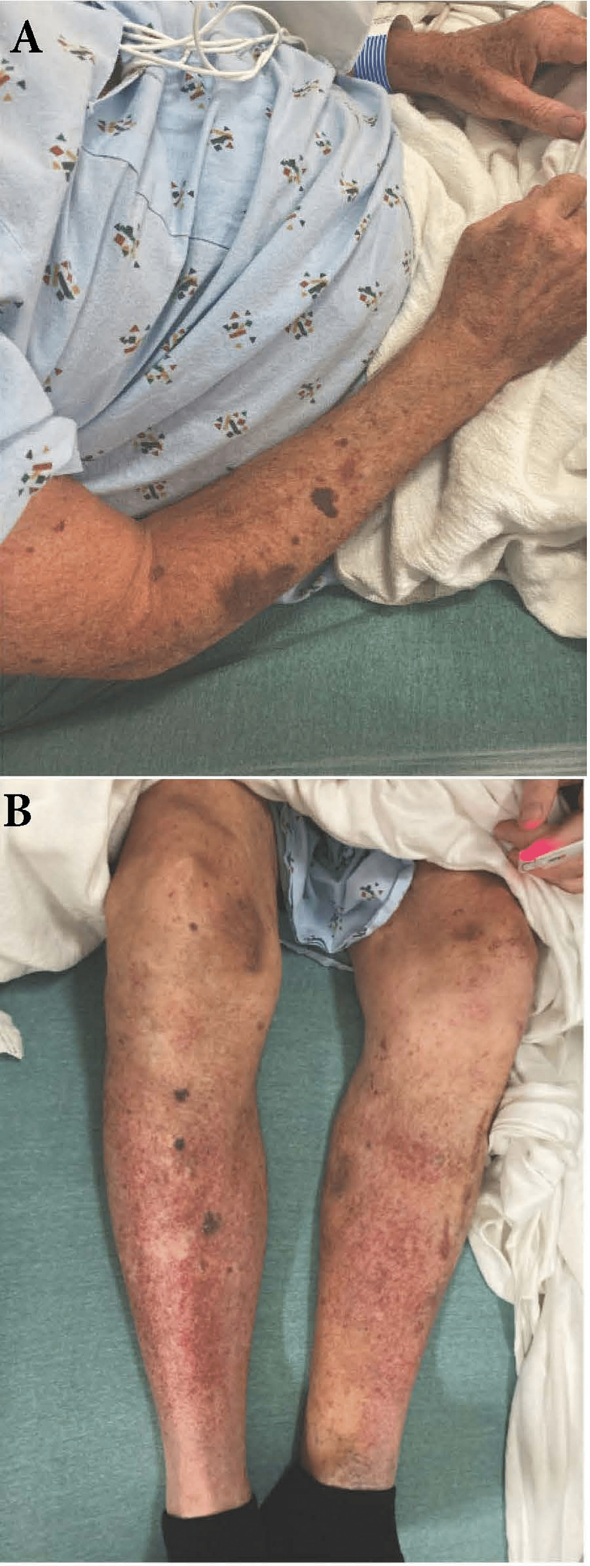
(A) and (B) Cutaneous manifestations on presentation.

The patient had been in his usual state of health until he developed a rapidly progressive mixed purpuric/petechial rash on his upper extremities and oral bleeding (Figure 1). He presented to his primary care provider (PCP) who performed a CBC that revealed severe thrombocytopenia (2,000 platelets, reference >150,000). A CBC from only three months prior had been normal, and all his prior CBCs had been hitherto unremarkable. As a result, he was referred to our tertiary care center for further management.

On a review of systems, he denied associated fevers, night sweats, or unintended weight loss. With regards to medications, he denied taking any over-the-counter drugs or supplements, confirming that the following were his only medications as prescribed by his PCP: Aspirin, Rosuvastatin, Zolpidem, Bupropion, Oxcarbazepine, Celecoxib, Tadalafil, Oxycodone, and Tamsulosin. His medical history was notable for *H. pylori *infection diagnosed on histology following an esophagogastroduodenoscopy (EGD) performed as a result of years of chronic dyspepsia. Although this diagnosis had been established two months prior, for unclear reasons, he had not undergone *H. pylori* eradication treatment. His other reported chronic conditions included an unprovoked deep venous thrombus (DVT) (for which he had completed a three-month course of apixaban six months prior), osteoarthritis, and hypercholesterolemia. Pertaining vaccination, he was up to date with age-appropriate vaccines, having received two doses of the COVID Pfizer vaccine (with the last dose received approximately 75 days prior) and the influenza vaccine (received six days prior to symptom onset). On social history interview, he denied any recent travel outside the United States and ever using illicit drugs and was in a monogamous relationship. 

His physical examination was notable for normal vital signs and remarkable for purpura on the bilateral upper extremities, a petechial rash on the bilateral lower extremities, and oral mucocutaneous lesions (not shown) (Figure 1). His neurologic and abdominal examinations were both unremarkable.

Investigations

To rule out pseudothrombocytopenia, a blood smear was examined and was notable for large platelets without clumps. Consumptive processes, such as TMA, microangiopathic hemolytic anemia (MAHA), and DIC, were ruled out based on the noted absence of schistocytes on the smear, normal clotting times, and normal levels of haptoglobin, fibrinogen, and LDH (Table 1). Viral etiologies frequently associated with thrombocytopenia were also investigated and ruled out (Table 2). The possibility of an underlying hematologic malignancy was rule out through peripheral flow cytometry, as our suspicion for an underlying lymphoma or leukemia was minimal, leading us to defer a bone marrow biopsy. Nutritional deficiencies associated with marrow suppression, for example, copper and vitamin B12 were also investigated and ruled out.

**Table 1:** Acute infectious viral panel. CMV, cytomegalovirus; EBV, Epstein Barr virus; HCV, hepatitis C virus; PCR, polymerase chain reaction

Virus	Result
HIV	Negative
CMV IgG	Negative
CMV IgM	Negative
COVID-19 PCR	Negative
EBV	Not detected
HCV	Negative
Acute hepatitis B panel	Negative

**Table 2:** Baseline laboratory values with indicators. N, normal; H, high; L, low; INR, international normalized ratio; APTT, activated partial thromboplastin time.

Parameter	Value	Reference range
Sodium	134 (below normal)	135-145
Potassium	3.7 (N)	3.5-5
Chloride	97 (N)	95-105
Bicarbonate	23 (N)	23-29
Urea Nitrogen	17 (N)	5-20
Creatinine	1.04(N)	0.8-1.2
Copper	102.3 (N)	62-140
Zinc	70.6 (N)	44-115
Vitamin B12	951 (N)	160-950
Folate	664 (H)	4.5-45.3
White Blood Cell Count	11.2 (N)	3.8-10.4
Hemoglobin	14 (N)	13.6-16.9
Hematocrit	40.3 (N)	40-50
Mean corpuscular volume	87.8 (N)	80-99
Platelets	4 × 10E3 (L)	150 × 10E3 to 450×10E3
INR	1 (N)	0.8-1.1
APTT	26.3 (N)	21-35

HITT antibody testing to investigate vaccine-induced Immune thrombotic thrombocytopenia (VITT) from the COVID-19 vaccine was also considered but was deferred because the patient was outside the window (4-42 days) stipulated by the American Society of Hematology (ASH) expert opinion [5].

Treatment and outcome

The patient was treated with 1 g IV methylprednisolone followed by one dose of 0.5 mg/kg of IV immunoglobulin (IVIG) on day 1 of admission. Additionally, per our hospital’s severe thrombocytopenia protocol, he underwent neurological exam by nursing every two hours.

Within 24 hours, his platelets improved to 24,000, and he was transitioned to a course of oral dexamethasone 40 mg for three days with further improvement in counts to 152,000. He was discharged with a steroid taper on day 3 and urgent outpatient oncology follow-up.

## Discussion

We hereby present a case of a 70-year-old man with a history of untreated *H. pylori *infection who developed ITP following the influenza vaccine. We suspect that the patient’s underlying untreated *H. pylori* infection and advanced age collectively increased his risk of this vaccine side effect. While *H. pylori *infection itself has been associated with ITP, the timeline in this case, specifically, suggests a proximal precipitant and implicates the vaccine: the patient had longstanding dyspepsia (dating at least two years per chart review) without associated thrombocytopenia.

It is critical to underscore that ITP following the influenza vaccine is rare: per the Vaccine Adverse Events Reporting System (VAERS) database [6], there were only 133 cases reported between 1990 and early 2022 in the United States [6]; additionally, there are only a handful of case reports in the literature [7-14]. These reports largely document experiences similar to our own, specifically about onset (the condition typically develops about four to seven days following vaccination). Among these studies, one report provides the most compelling evidence of an association, documenting ITP recurrence on three separate occasions following the influenza vaccine within the same patient [8].

Considering the rarity of this side effect and the limited evidence linking it within the literature (mostly based on case reports), the advantages of immunization outweigh the potential risks of not receiving the vaccination to prevent ITP. Nonetheless, the risk of this potentially fatal side effect is not zero. As a result, identifying patients with underlying risk factors for ITP (and addressing those factors) is not only warranted but also in alignment with the principle of beneficence in medicine.

## Conclusions

The influenza vaccine carries a low risk of ITP. But underlying comorbidities, such as untreated *H. pylori* infection, might interact with additional patient factors, such as older age, to amplify this risk in some. Well-designed studies are needed to identify patients at increased risk of developing this adverse outcome and ultimately develop preemptive interventions.
